# In vivo bioluminescence imaging and histopathopathologic analysis reveal distinct roles for resident and recruited immune effector cells in defense against invasive aspergillosis

**DOI:** 10.1186/1471-2180-10-105

**Published:** 2010-04-08

**Authors:** Oumaïma Ibrahim-Granet, Grégory Jouvion, Tobias M Hohl, Sabrina Droin-Bergère, François Philippart, Oh Yoen Kim, Minou Adib-Conquy, Reto Schwendener, Jean-Marc Cavaillon, Matthias Brock

**Affiliations:** 1 Unité de Recherche Cytokines & Inflammation, Institut Pasteur Paris France; 2 Unité de Recherche et d'Expertise Histotechnologie et Pathologie, Institut Pasteur Paris France; 3 Infectious Disease Service, Memorial Sloan-Kettering Cancer Center, New York, NY, USA; 4 Laboratory of Liposome Research, Institute of Molecular Cancer Research, University of Zurich, Zurich, Switzerland; 5 Leibniz Institute for Natural Product Research and Infection Biology, Hans Knoell Institute, Junior Research Group Microbial Biochemistry and Physiology, Jena, Germany; 6 Current address: Fred Hutchinson Cancer Research Center, Seattle, WA, USA

## Abstract

**Background:**

Invasive aspergillosis (IA) is a major cause of infectious morbidity and mortality in immune compromised patients. Studies on the pathogenesis of IA have been limited by the difficulty to monitor disease progression in real-time. For real-time monitoring of the infection, we recently engineered a bioluminescent *A. fumigatus *strain.

**Results:**

In this study, we demonstrate that bioluminescence imaging can track the progression of IA at different anatomic locations in a murine model of disease that recapitulates the natural route of infection. To define the temporal and functional requirements of distinct innate immune cellular subsets in host defense against respiratory *A. fumigatus *infection, we examined the development and progression of IA using bioluminescence imaging and histopathologic analysis in mice with four different types of pharmacologic or numeric defects in innate immune function that target resident and recruited phagocyte subsets. While bioluminescence imaging can track the progression and location of invasive disease in vivo, signals can be attenuated by severe inflammation and associated tissue hypoxia. However, especially under non-inflammatory conditions, such as cyclophosphamide treatment, an increasing bioluminescence signal reflects the increasing biomass of alive fungal cells.

**Conclusions:**

Imaging studies allowed an in vivo correlation between the onset, peak, and kinetics of hyphal tissue invasion from the lung under conditions of functional or numeric inactivation of phagocytes and sheds light on the germination speed of conidia under the different immunosuppression regimens. Conditions of high inflammation -either mediated by neutrophil influx under corticosteroid treatment or by monocytes recruited during antibody-mediated depletion of neutrophils- were associated with rapid conidial germination and caused an early rise in bioluminescence post-infection. In contrast, 80% alveolar macrophage depletion failed to trigger a bioluminescent signal, consistent with the notion that neutrophil recruitment is essential for early host defense, while alveolar macrophage depletion can be functionally compensated.

## Background

Morbidity and mortality caused by invasive *Aspergillus *infections are increasing due to an expansion in the number of patients receiving potent myeloablative and immunosuppressive regimens for transplantation and the treatment of malignancy and autoimmune disorders [1,2]. Patients with hematologic malignancies and recipients of allogeneic hematopoietic stem cell transplants (HSCT) belong to high-risk groups for invasive aspergillosis (IA).

IA is the most common invasive mould infection in immunocompromised patients. Although neutropenia following the conditioning regimen remains an important risk factor for IA in the early post-transplant period, most cases of IA in allogeneic HSCT recipients occur after neutrophil recovery in the setting of potent immunosuppressive therapy for graft-versus-host disease (GVHD). This treatment of GVHD in the late post-transplant period with corticosteroids and potent immunosuppressive therapy contributes to the risk for IA [3-7].

In immune competent hosts, pulmonary alveolar macrophages (AM) coordinate the early inflammatory response and ingest and kill the inhaled conidia [8,9]. Besides ingesting inhaled conidia, AMs are believed to play a key role in orchestrating the inflammatory response to *A. fumigatus*. Pattern recognition receptors (PRR) on AM recognize specific fungal cell wall motifs displayed during the conidial and hyphal stages and produce cytokines and chemokines that stimulate neutrophil recruitment and subsequent antigen-specific immunity. Recent studies have demonstrated the key role of PRR in regulating innate and antigen-dependent immunity in response to fungal infections [10,11]. For instance, β-glucan that is exposed on the surface of *Aspergillus *germinating conidia and hyphal cells (but not resting conidia) is recognized by the C-type lectin, dectin-1 [12-14].

In addition to AMs, other innate immune cells that include neutrophils, monocytes and NK T cells have important antifungal effector roles. The critical role of neutrophils has been substantiated by the high risk of IA in patients who have severe and prolonged neutropenia and the lethal course of IA in neutropenic murine models [15].

Although the past few years have witnessed advances in our understanding of the pathophysiology of IA, our understanding of the disease process and the host response has been hampered by the inability to follow in vivo fungal growth and dissemination in real time.

We recently generated a bioluminescent *A. fumigatus *strain, which constitutively expresses the luciferase from *Photinus pyralis *under control of the glyceraldehyde-3-phosphate dehydrogenase promoter. We showed that the bioluminescence of this strain correlated well with fungal biomass under in vitro conditions and demonstrated that using bioluminescence imaging enables researchers to monitor the onset of pulmonary IA in corticosteroid-treated mice [16].

In the present study we applied bioluminescence imaging to an animal model of IA by using different immunosuppression regimens that affect either AM and/or neutrophil number or function. The primary aim of this study was to evaluate the suitability of in vivo and ex vivo bioluminescence imaging to monitor the development of invasive aspergillosis. Bioluminescence imaging was combined with quantitative fungal DNA determination, histopathologic and morphometric analysis of infected lung tissues to assess the fungal burden and progression of IA, the timing and pattern of extrapulmonary dissemination, and the development of inflammatory cell infiltrates in the lung. Our results suggest that neutrophils, rather than AM, play an indispensable role in host defense against *A. fumigatus*.

## Results

### Pathogenesis of invasive aspergillosis following different immunosuppression regimens

**Figure 1:**
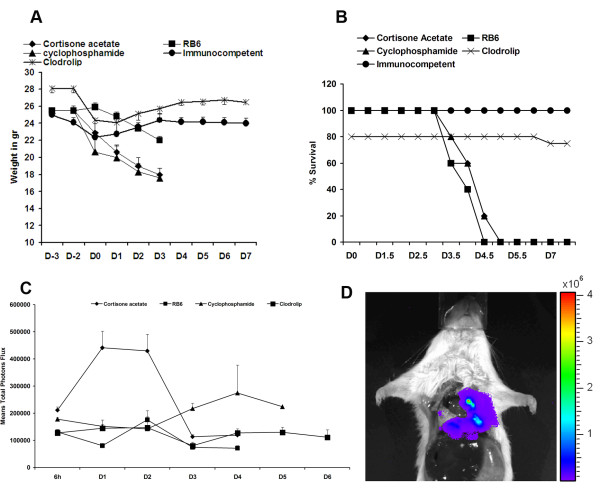
**Clodrolip treated mice are not susceptible to *A. fumigatus *intranasal infection**. In each experiment, groups of 5 mice were treated either with cortisone acetate, cyclophosphamide, RB6-8C5 antibody, or clodrolip prior to intranasal infection with 2 × 10^6 ^conidia of the luminescent *A. fumigatus *strain C3. Untreated infected mice are designated as immnocompetent (IC). Weight loss and survival were monitored for 8 days (A and B). (C): Time response study of luminescence emission from chest region 10 min after intraperitoneal injection of D-luciferin. Light emission from live animals was recorded for 5 min. Each point represents the average from 3 independent experiments of the total photon flux measured from a defined thoracic region from each individual animal of the respective cohort (5 mice). (D): Light emission from the lung of a dead animal immunosuppressed with cortisone acetate following direct injection of D-luciferin. A total photon flux/second of 3.744 × 10^6 ^has been measured using the living image software 3.1 after 1 min exposure.

Different immunosupression regimens were used to study their impact on murine survival, the development of invasive aspergillosis (IA), and on fungal growth and dissemination, using the bioluminescent *A. fumigatus *strain C3 [16]. Immune competent mice manifested a transient weight loss on the day of infection (Figure 1A) and uniformly survived the infection (Figure 1B). As expected, mice treated with the alkylating agent cyclophosphamide or the glucocorticoid cortisone acetate died within five days after infection (Figure 1B) and progressive infection was accompanied by ongoing weight loss (Figure 1A). Both treatments are frequently used for testing the virulence of *A. fumigatus *and these results confirmed the virulence of bioluminescent strain C3 in different infection models.

Neutrophils were depleted by using the monoclonal antibody RB6-8C5, which binds the myeloid differentiation antigen Gr-1 and leads to neutropenia lasting for three to four days at the dose administered in our experiments [17]. In agreement with prior studies, transient neutropenia was sufficient to cause lethal pulmonary aspergillosis (Figure 1B) [17]. However, weight loss of mice treated with RB6-8C5 was less pronounced than observed with the other immunosuppressive regimens (Figure 1A).

We also targeted resident alveolar macrophages by intranasal instillation of liposomes containing clodronate (clodrolip). Phagocytosis of clodrolip leads to an intracellular accumulation of clodronate and the induction of macrophage apoptosis [18]. To confirm that the observed effects were not due to the instillation with liposomes, empty liposomes served as a control. The 20% loss of treated mice shown in Figure 1A is due to the accidental death of one mouse that displayed pulmonary haemorrhages after drug administration, at necropsy. After infection, none of the mice treated with clodrolip showed severe signs of illness and weight loss was transient (Figure 1A and 1B).

### Bioluminescence imaging of infected mice

To understand the specific impact of each immunosuppression regimen on fungal growth, we performed in vivo bioluminescence measurements in different infected cohorts of mice using *A. fumigatus *strain C3. Subsequently, we performed histopathologic analyses to correlate the light emission pattern with fungal invasion and immune effector cell recruitment.

Figure 1C shows a time response of the quantification of the luminescence from the thorax of animals treated with the different immunosuppressive agents. As previously observed, light emission peaked between day one and two post-infection in cortisone acetate-treated mice. A peak in the bioluminescence signal at day two post-infection was observed in mice that received the RB6-8C5 antibody. However, the thoracic signal intensity was much weaker in RB6-8C5-treated mice than in cortisone acetate-treated mice and hardly exceeded the background intensity. Despite the low signal intensity, all mice died four or five days post-infection. Cyclophosphamide treatment, in contrast, induced a more gradual rise in bioluminescence on day three post-infection. The signal intensity continued to increase and remained at a high level until death of the animals at day five post-infection, implying that biomass formation may correlate best with bioluminescence development under this immunosuppresive treatment. Mice treated with clodrolip did not show overt signs of disease and the bioluminescence signal remained near the imaging threshold of approximately 5 × 10^4 ^- 1 × 10^5 ^total photon flux per second. This result suggested that despite AM depletion, no significant hyphal growth occurred after clodrolip treatment.

In summary, these results suggest that the rapid increase in bioluminescence, observed in cortisone acetate-treated mice in particular, but also in RB6-8C5-treated mice, reflects early conidial germination post-infection.

### Correlation of bioluminescence signals with fungal burden in infected mouse lungs

**Figure 2:**
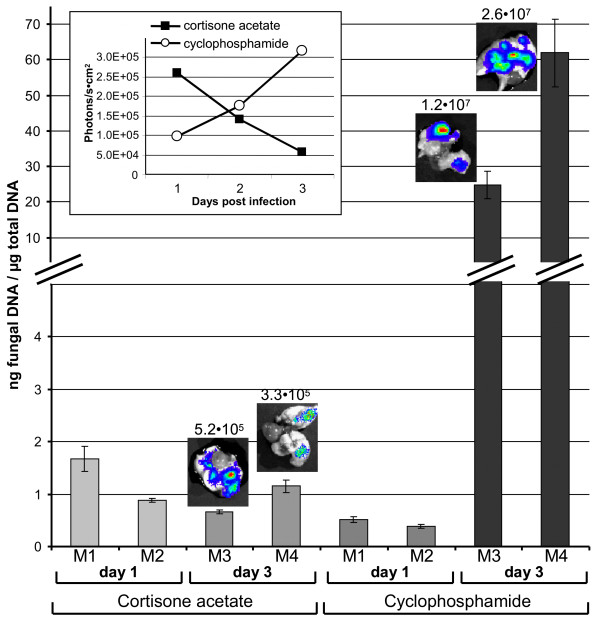
**Quantitative real-time PCR of fungal DNA enables the correlation between fungal burden and bioluminescence signals**. Mice were immunosuppressed either with cortisone acetate or cyclophosphamide. Two mice from each group were sacrificed at day one and the other two animals from each group at day three after infection. An uninfected mouse was used as a negative control and revealed no signal in the qRT-PCR and is, therefore, omitted from the graph. The bars represent the amount of fungal DNA per microgram of total DNA isolated from the infected tissues with standard deviations from six data points for each individual animal. The two animals investigated for each time point and immunosuppression regimen show the general tendency that at day one after infection the cortisone acetate treated animals show a higher burden than the cyclophosphamide treated animals. Three days after infection, the burden with alive fungal cells seems to stay rather constant under the coticosteroid treatment, but strongly increases under the regimen with cyclophosphamide. The inlet shows the time response of bioluminescence from alive animals with high values for the cortisone acetate treated mice early after infection followed by a decline of the signal intensity at later time points. Under cyclophosphamide regimen the bioluminescence steadily increases. The small photographs above the bars from mice sacrificed at day three show the explanted lungs with an overlay of the emitted light intensities. Numbers above the photographs give the photons/s × cm^2^. The high bioluminescence from lungs explanted from cyclophosphamide treated animals reflects the higher burden with living fungal cells as determined by the fungal DNA quantification.

To correlate our assumption concerning the germination speed of conidia with the bioluminescence signal intensities under different immunosuppression regimens, we performed additional experiments on mice immunosuppressed either with cortisone acetate or cyclophosphamide. Mice were infected with the bioluminescent strain C3 and sacrificed after bioluminescence monitoring on day one or three after infection. Lungs of these mice were used to determine the fungal burden by quantification of the fungal DNA among the total DNA isolated from lung tissues (Figure 2). As observed before (Figure 1), the development of bioluminescence was most strongly pronounced at early time points under cortisone acetate treatment, but declined at later stages of infection. Contrarily, under cyclophosphamide treatment the bioluminescence signal was hardly detectable one day after infection, but steadily increased at later time points (Figure 2, inlet). As assumed, the amount of fungal DNA detected one day after infection in cortisone acetate treated animals was generally higher than that of cyclophosphamide treated animals at the same time point, confirming an increased early germination rate of conidia under corticosteroid treatment. Surprisingly, the quantity of fungal DNA stayed rather constant under the corticosteroid regimen. This implies that the immune response under this treatment either prohibits further growth of hyphae or even kills fungal cells, which could explain the decrease in the bioluminescence signal. However, lungs explanted from mice sacrificed at day three still showed significant luminescence (Figure 1D and 2). Therefore, we assume that, besides reducing the expansion of fungal mycelium through the lung tissue, neutrophils cause extensive tissue destruction leading to tissue hypoxia, which could attenuate the bioluminescence signal. Oxygen is an essential substrate for firefly luciferase activity and an oxygen saturation below 5% significantly decreases light emission [19].

In contrast, treatment with the cytostatic drug cyclophosphamide prevents the recruitment of immune effector cells to the side of infection. Therefore, despite a retarded germination of conidia, fungal hyphae stay alive, which is well visualized by the massive increase in fungal DNA determined at the late stage of infection (Figure 2). In agreement, the bioluminescence steadily increased under this regimen and explanted lungs show a 50 - 100 times higher light emission than observed under corticosteroid treatment. This result shows that bioluminescence measurements and DNA quantification correlate best under the cyclophophamide regimen. Although the bioluminescence readout does not correlate linearily with the fungal burden as measured by qRT-PCR, the general tendency of increasing and decreasing fungal burden as well as the impact of the inflammatory response seems well reflected by bioluminescence imaging.

### Impact of immunosuppression regimens on the inflammatory response

In order to correlate survival curves, weight loss, fungal burden from DNA quantification and bioluminescence with histopathological findings, additional experiments were performed, in which mice were sacrificed one day (early) and three days (late) post infection. For the clodrolip condition, mice were sacrificed eight days after infection to assess any later effect of treatment on mice survival. Lungs were removed, and thin sections were studied for the evaluation of the recruitment of immune effector cell lineages and fungal tissue invasion.

### Clodrolip treatment

Lung instillation with clodrolip was expected to reduce the number of AM, which are generally denoted as the first cellular line of host innate immune defense through phagocytosis and killing of inhaled conidia. To confirm the reduction in the number of AM, the BAL fluid of non-infected mice were sampled two days after intranasal administration of clodrolip or liposomes, respectively. Flow cytometry was used to quantify the number of AM within the BAL fluid. The clodrolip treatment resulted in a numeric depletion of 60% of AM (8.30 × 10^4 ^± 1 × 10^4 ^versus 2.03 × 10^5 ^± 1.8 × 10^4^) when compared to control liposome treated animals (p < 0.05). Furthermore, the viability of the residual AM subset was only 50% as evaluated by trypan blue staining. Taken together, clodrolip treatment depleted or resulted in the death of 80% of AM compared to control mice.

**Figure 3:**
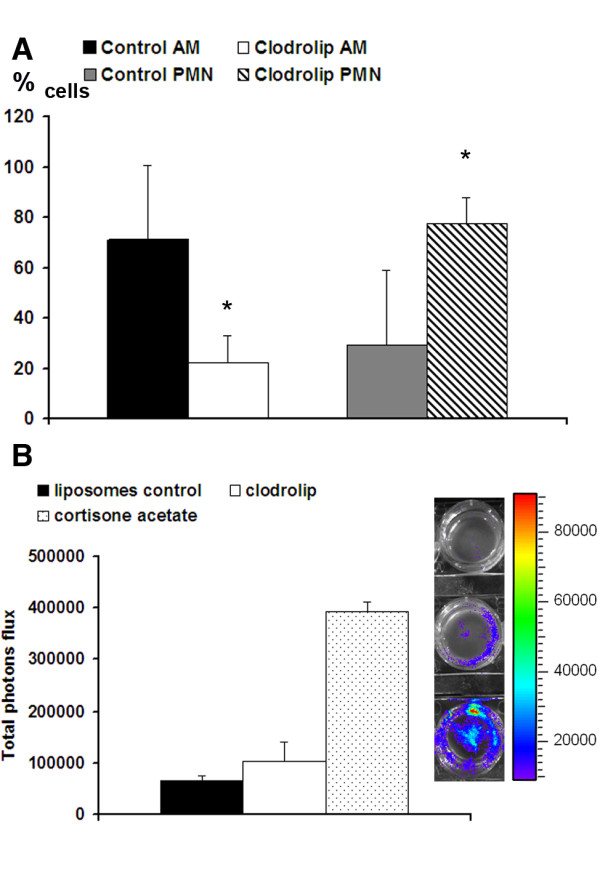
**Neutrophil recruitment inhibits the conidial germination in alveolar macrophages-depleted mice one day after infection**. (A): Alveolar macrophage and neutrophil populations were counted in BAL fluids one day after infection of mice treated with the liposome control and clodrolip. N = 5 mice per group. One of three independent experiments is shown. * denotes a p-value < 0.05. (B): Light emission in BAL-fluids one day after infection of mice treated with liposome control (upper cell well), clodrolip (middle cell well) and cortisone acetate (lower cell well). BAL cells were collected by cytospin centrifugation using labtek chamber slides. D-luciferin was incorporated to the medium and luminescence acquired after 10 min with the IVIS 100 system. The graph shows the total luminescence evaluated by using the living image software 3.1.

When the cell populations in BAL were evaluated one day post-infection, we noted a 3.2-fold decrease (22 ± 11 versus 71 ± 28%) in the concentration of AM and a 2.6-fold increase (77.5 ± 10 versus 29 ± 28%) in the neutrophil concentration in clodrolip-treated mice compared to control liposome-treated mice (Figure 3A). Thus, AM depletion did not result in an apparent defect but rather in airway neutrophil recruitment 24 h post-infection.

Furthermore, we performed an evaluation of the luminescence in the BAL one day after infection, comparing clodrolip versus liposomes (control) or cortisone acetate treated mice. Cortisone acetate was used as a positive control for fungal germination within the lung tissue, because we previously showed that cortisone acetate inhibits the killing capacity of AM and resulted in the germination of conidia even one day after infection [20,21]. Mice treated with clodrolip had a fourfold lower BAL luminescence signal than cortisone actetate-treated mice (102000 ± 37000 versus 394000 ± 19500 photons flux) (Figure 3B), consistent with the finding that preserved airway neutrophil recruitment under these conditions can inhibit the conidial germination. However, although not significantly different, the signal in the BAL from clodrolip treated mice was higher than that of liposome treated control mice (102000 ± 37000 versus 66300 ± 19500). Nevertheless, germination and mycelium formation was inhibited in AM-depleted mice as confirmed by lung histopathology analyses performed one and eight days post infection (see below).

#### Neutrophils may act as the first line of defense against conidia

**Figure 4:**
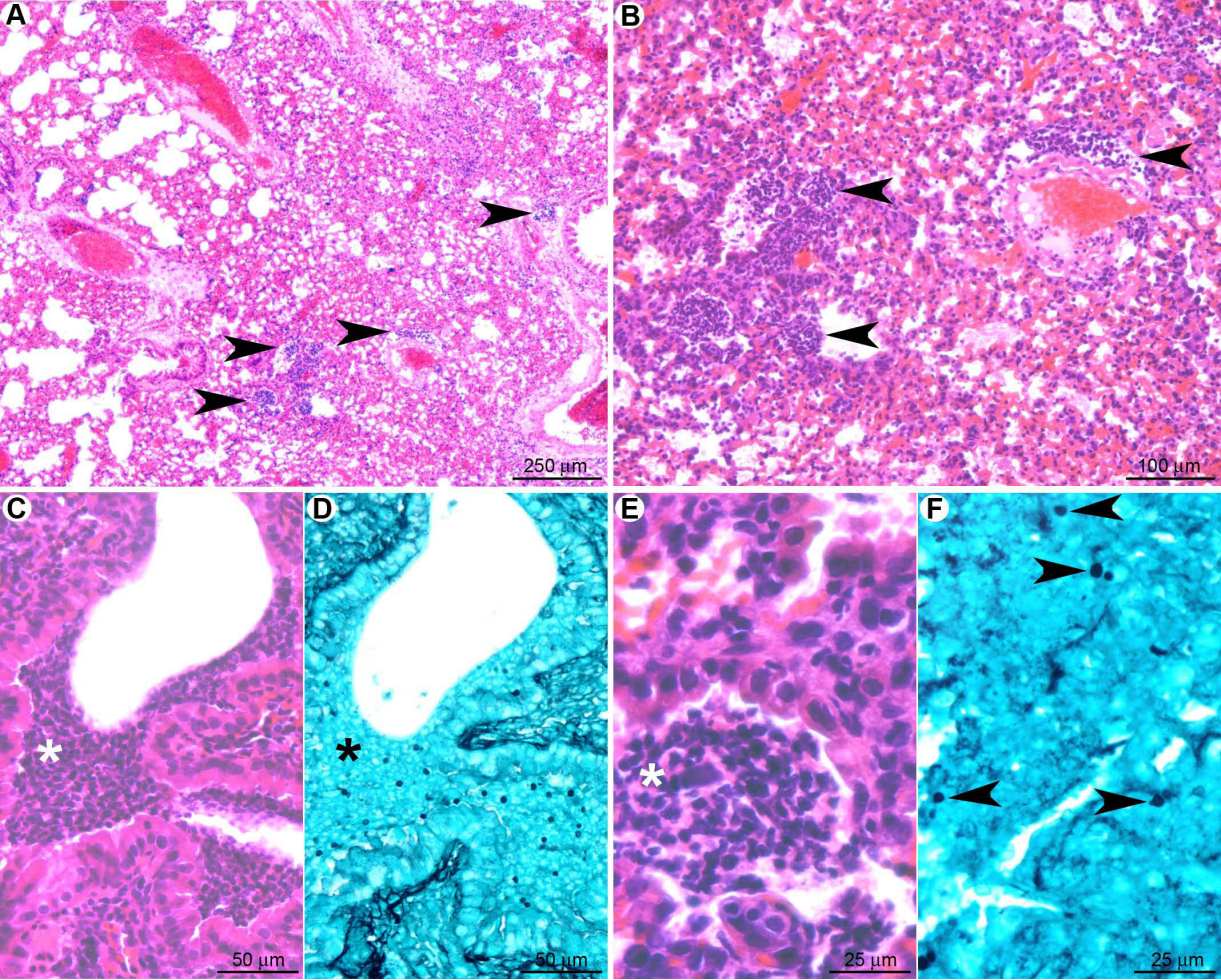
**At the early stage of pulmonary colonisation, neutrophil influx limits fungal germination after clodrolip treatment**. (A): Multifocal inflammatory lesion centred on bronchi/bronchioles and blood vessels (arrowheads). (B): Inflammatory cells observed in alveolar spaces (arrowheads). (C, E): Inflammatory infiltrates containing fragmented neutrophils (suppuration, white stars). (D, F): In the inflammatory infiltrates (black star) only non-germinated conidia (arrowheads) were observed. *A, B, C, E: HE staining; D, F: GMS staining*.

One day post-infection, the lungs of clodrolip-treated mice contained multifocal lesions (Figure 4A) characterised by scattered hemorrhagic foci associated with small (surface < 200 μm^2^) perivascular, peribronchiolar, or intra-bronchiolar/alveolar inflammatory infiltrates (Figure 4B). At this stage, few macrophages were detected, which implies that alveolar macrophage depletion was not compensated by massive monocyte recruitment at day one after infection. The cellular infiltrates contained mostly karyorrhectic (i.e. fragmented) neutrophils (Figure 4C, E), embedded in a necrotic material associated with extravasated erythrocytes. Clusters of non-germinated conidia were observed in the neutrophilic infiltrates (Figure 4D, F).

**Figure 5:**
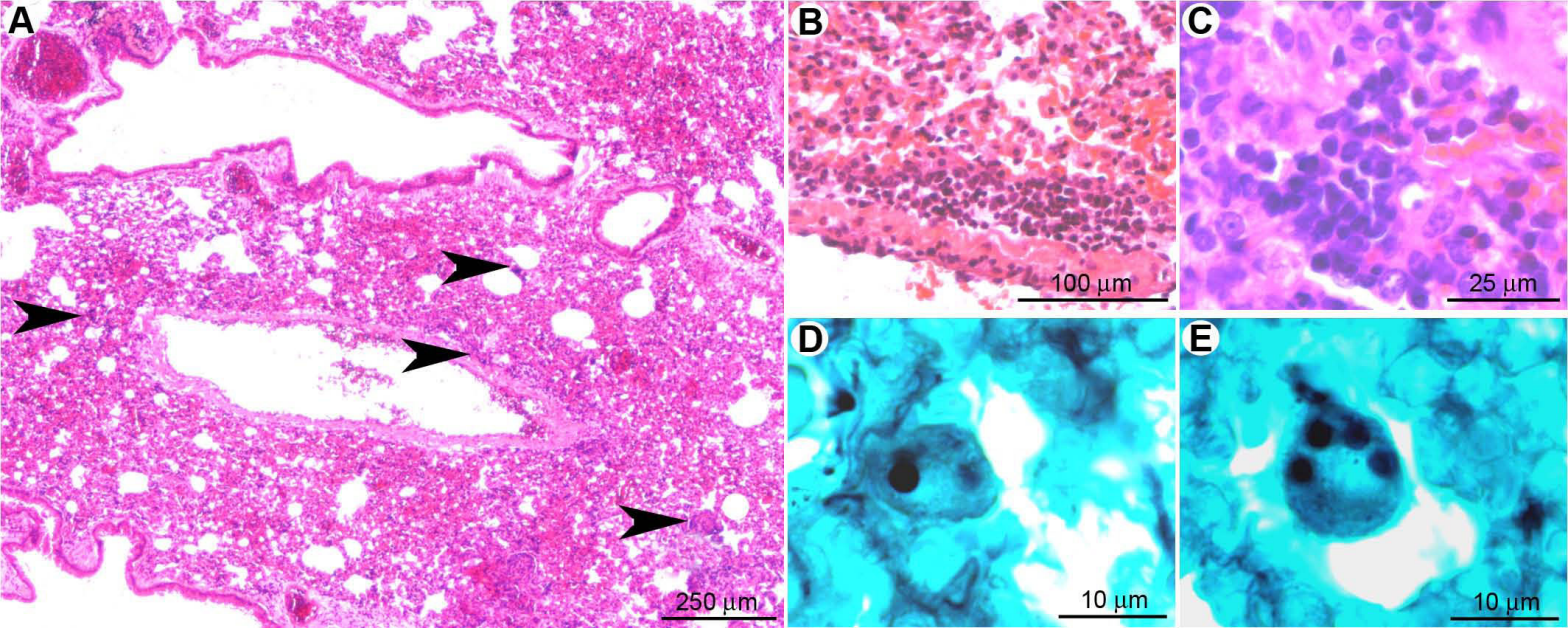
**Eight days post-inoculation, hyphal growth was not observed in clodrolip treated mice**. (A): At low magnification, very few lesions (hemorrhages and small inflammatory infiltrates) were observed (arrowheads). (B, C): Inflammatory infiltrates were characterised by perivascular and peribronchiolar accumulation of lymphocytes and plasma cells. (D, E): A small number of non-germinated conidia, located in the cytoplasm of alveolar macrophages were observed. *A, B, C: HE staining; D, E: GMS staining*.

Eight days post-infection, the lungs of euthanized mice displayed inflammatory lesions (Figure 5A) characterised by multifocal hemorrhages and peri-vascular/bronchiolar lymphocyte and plasma cell infiltration (Figure 5B, C). Very few non-germinated conidia were detected in the cytoplasm of macrophages located in alveolar spaces (Figure 5D, E). At this stage, morphometric analysis revealed that the total surface of the inflammatory cell infiltrates was 2.9 ± 1.7% of the total lung parenchymal surface on the histologic sections, in comparison to 1.8 ± 1.0% at day one after infection (Table 1). These data indicate that, at early post-infection time points, neutrophils have supplanted AM as a first line of host defense, leading to the destruction and inactivation of conidia prior to germination and hyphae formation. The observed absence of fungal hyphae under these conditions correlated with the inability to detect an increase of the bioluminescence signal.

**Table 1:** Comparison between the lesion profiles in the different immunosuppressive conditions.

		Lesions			Recruted Inflammatory Cells		Fungi	
		**Proportion of inflammatory infiltrate surface**	**Major Localisation**	**Necrosis**	**Neutrophiles**	**Macrophages**	**Conidia**	**Hyphae**

**Chlodrolip**	**Early** 1 day PI	1.8 ± 1.0%	Random distribution	-	++	+/-	++	-

	**Late** 8 days PI	2.9 ± 1.7%	Perivascular Peribronchiolar	-	-	+	+	-

**Cortisone Acetate**								

	**Early** 1 day PI	3.8 ± 2.0%	Alveoli	++	++	+/-	++	+/-
	**Late** 3 days PI	11.2 ± 1.9%	Bronchi Bronchioles	+++	+++	++	++	+++

**RB6-8C5**								

	**Early** 1 day PI	1.9 ± 0.5%	Random distribution	+/-	-	+/-	+	-
	**Late** 3 days PI	18.9 ± 2.8%	Bronchi Bronchioles	+++	-	+++	++	+++

**Cyclophosphamide**								

	**Early** 1 day PI	No infiltrate	Bronchioles	+/-	+/-	-	+/-	-
	**Late** 3 days PI	No infiltrate	All structures	++++	-	-	+	++++

A group of five mice was studied at each time point, for each condition. The lesions were very similar between the different animals in a same group (PI: post-infection). A group of three mice was studied for the measurement of the surface of inflammatory cell infiltrates in tissue sections (morphometric analysis, 22 to 40 microscopic fields covering an entire lung section for each animal).

- *none, +/- minimal, + mild, ++ moderate, +++ marked, ++++ severe*

### Cortisone acetate treatment versus the combination of cortisone acetate and clodrolip

**Figure 6:**
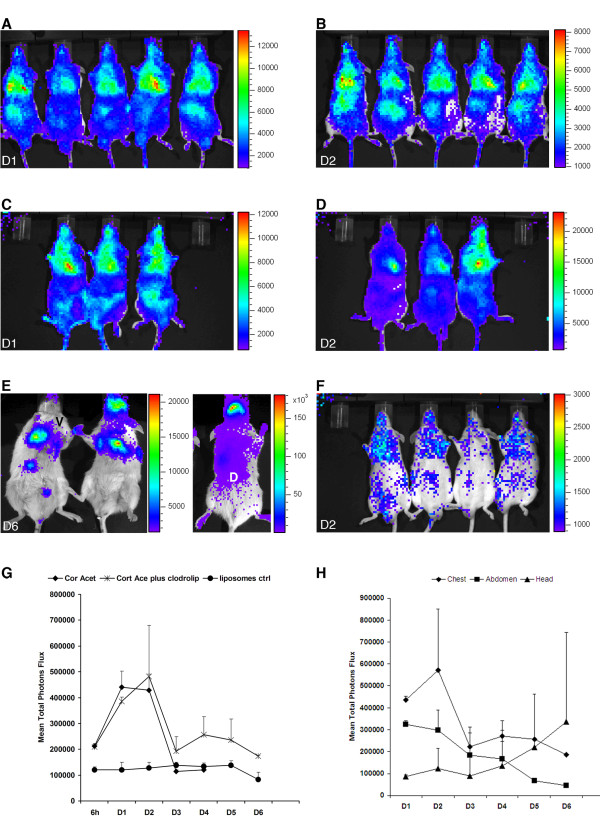
**Bioluminescence enables detection of thoracic and extra thoracic signals in cortisone acetate treated mice**. (A): Time response study of luminescence emission from mice immunosuppressed either with cortisone acetate (A, B) or with a combination of cortisone acetate and clodrolip (C-E). Mice were intranasally infected with 2 × 10^6 ^conidia. A cohort of 10 mice received liposomes as a control prior to infection (F). Images of day one (D1) and two (D2) post-infection are shown. Luminescence was monitored 10 min after intraperitoneal injection of D-luciferin. Images from ventral (V) and dorsal (D) views of the sinus region, six days after infection (D6) of mice treated with both, cortisone acetate and clodrolip, are shown (E). The graph in (G) represents the average of the total photon flux measured from a defined thoracic region from each individual animal of the respective cohort. (H): Time course of total luminescence from chest, abdomen and head regions from animals receiving the combination of cortisone acetate and clodrolip.

As shown in Figure 1C, 2 (inlet) and 6, mice treated with cortisone acetate (Figure 6A-B) or the combination of cortisone acetate and clodrolip (Figure 6C-D) displayed the highest peak of lung luminescence between day one and day two post infection. Both treatment groups experienced 100% mortality five to six days after infection. In addition to the thoracic region, a significant luminescence was observed from the abdomen of all infected mice. However, the abdominal signal declined rapidly and therefore was unlikely to result from fungal dissemination. This was confirmed in histology, by the absence of fungal CFU from the liver, spleen, stomach, and kidneys (data not shown). Therefore, it is likely that some conidia were swallowed and maintained for some time within the intestinal tract without manifestation of an infection. In contrast, a luminescence signal from the sinus regions has been observed in 20% of infected mice. This signal steadily increased and peaked during the terminal survival phase of illness (Figure 6E). In parallel with the bioluminescence increase from the sinus region, these infected mice became ataxic and displayed a disturbance in equilibrium. These data demonstrate that bioluminescence imaging can detect signals from extrathoracic sites.

#### Neutrophils encircle *A. fumigatus* conidia and limit their infiltrative potential, but fail to prevent their germination under corticosteroid-treatment

**Figure 7:**
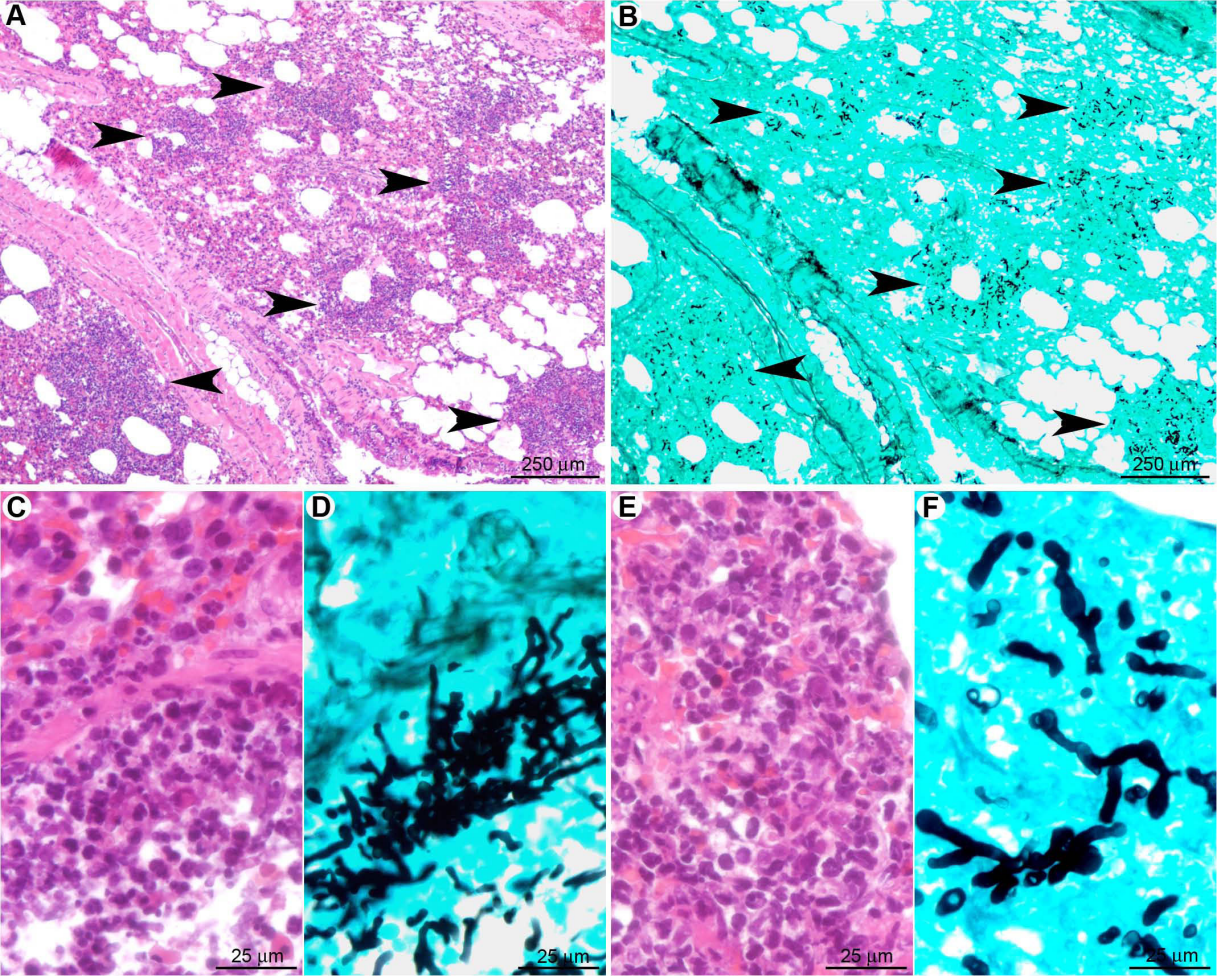
**The cortisone acetate mediated neutrophil infiltration did not prevent conidia germination even one day after infection**. (A): Multifocal inflammatory lesion extending from bronchi/bronchioles to alveoli (arrowheads). (B): Numerous fungal cells can be detected in the inflammatory infiltrates (arrowheads). (C, E): In the bronchioles (C) as well as in the alveoli (E), inflammatory infiltrates contained numerous neutrophils, which were very often fragmented (suppuration). (D, F): Bronchiolar spaces contained mature hyphae (D) in contrast to alveolar spaces that contained poorly mature hyphae and early germinating conidia (F). *A, C, E: HE staining; B, D, F: GMS staining*.

For histopathological analysis, five mice were sacrificed one day post-infection to visualise fungal outgrowth and the immune response in the early phase of infection. The late phase of infection, at which all the five studied mice displayed strong clinical signs of illness, was studied in lung sections derived from dying animals 3-4 days post-infection. Lung histopathology at one day after infection revealed multifocal inflammatory lesions mostly centred on alveoli but also involving some bronchial/bronchiolar spaces (Figure 7A). They were characterised by small to large infiltrates (surface up to 500 μm^2^) of neutrophils that were often karyorrhectic and associated with the necrosis of the overlying epithelium (Figure 7C, E). The total surface of inflammatory infiltrates was 3.8 ± 2.0% of the total lung parenchyma surface (Table 1). Germinating conidia and hyphae were diffusely observed in bronchiolar and alveolar spaces, as well as in the interalveolar septae (Figure 7B), but they displayed different maturation stages. Bronchiolar spaces contained mature septated hyphae (Figure 7D), in contrast to alveolar spaces, where only early germinating conidia and short hyphal germlings were detected (Figure 7F). These experiments confirm the data obtained from the quantification of fungal DNA within the infected tissues, which implied that conidia are rapidly germinating under cortisone acetate treatment.

In comparison to clodrolip-treated mice (Table 1), cortisone acetate-treated mice exhibited a higher and more severe level of pulmonary parenchyma destruction, and conidia and hyphae were at a more advanced stage of maturation.

**Figure 8:**
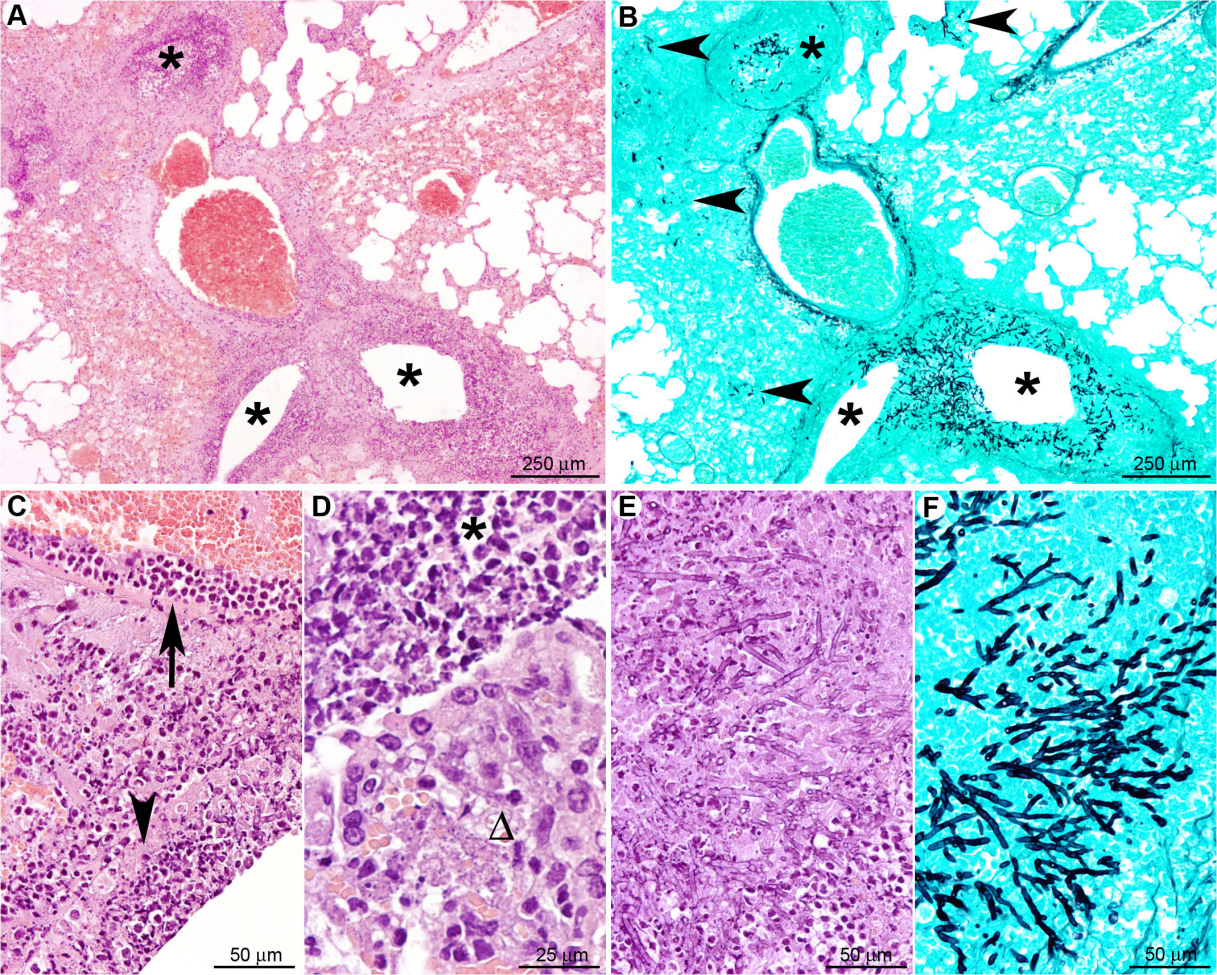
**Despite strong infiltration of neutrophils under cortisone acetate treatment, growth of the fungus in bronchiolar and alveolar spaces is not prevented in the late stage of infection**. (A): Multifocal to coalescing inflammatory lesion centred on bronchioles (black stars) and extending to alveoli and blood vessels. (B): Mycelium growing mainly in the bronchiolar spaces (black stars), but also extending to alveoli (arrowheads). (C): Lesions displayed a concentric organisation: in the centre, neutrophils accumulated and infiltrated bronchioles (arrowhead) and blood vessel walls (arrow). (D): Neutrophils (black star) were circled by a peripheral rim of activated macrophages (Δ). (E, F): Fungi displayed a high infiltrative potential, extending from bronchiolar spaces to alveoli. *A, C, D, E: HE staining; B, F: GMS staining*.

Three days after infection (Figure 8), pulmonary inflammatory lesions within the corticosteroid-treated group were multifocal, centred on bronchi/bronchioles but secondarily extending to alveoli and blood vessels (veins and arteries), and displayed a concentric organisation (Figure 8A). In the centre of the inflammatory lesions, bronchiolar, alveolar and vascular spaces were infiltrated mostly by karyorrhectic neutrophils (Figure 8C, E). Neutrophils were circled by a peripheral rim of activated macrophages (epithelioid cells): pyogranulomatous lesion (Figure 8D). This was the only condition where pyogranulomatous lesions were observed and all the five mice of the studied group displayed similar lesions (nature and severity). The surface of these pyogranulomatous lesions was up to 1,370 μm^2^; the general inflammatory lesion filled 11.2 ± 1.9% of the total parenchyma surface (Table 1), indicating ongoing tissue destruction under cortisone acetate treatment. Numerous septated hyphae were detected in bronchial/bronchiolar spaces, but also infiltrating bronchiolar walls and spreading to peripheral alveoli (Figure 8B, F). Although histopathology indicates an increase in fungal biomass at the late stage of infection, a significant proportion of fungal cells might have been killed by neutrophil attack. This assumption is supported by the determination of the fungal burden by quantitative real-time PCR (Figure 2). Although this investigation was only performed on two animals for each time point and immunosuppression regimen, this analysis indicated that the number of living fungal cells does not seem to increase, since the amount of fungal DNA remains rather constant when compared to the early time point. Additionally, the massively observed tissue destruction indeed might cause hypoxic conditions accompanied by a decrease of light emission from lung tissues of corticosteroid treated mice.

The same pattern of severe lesions was observed after the clodrolip/cortisone acetate treatment (data not shown). Therefore, depletion of alveolar macrophages does not exhibit additional effects on the development of invasive aspergillosis in the presence of cortisone acetate.

**Figure 9:**
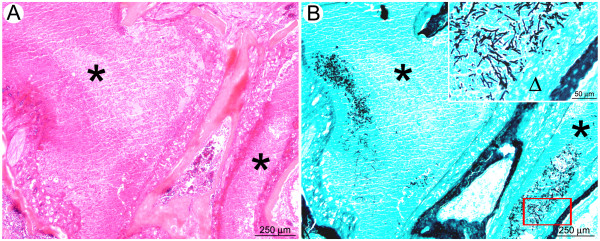
**After intranasal inoculation, mice treated by cortisone acetate could develop a suppurative sinusitis**. (A): The nasal sinus cavities were filled by a suppurative exudate containing fragmented neutrophils (black stars). (B): A high number of intralesional mature hyphae were detected in the exudate as well as along the nasal sinus epithelium (inlay, Δ).

Histopathological analysis from the sinus regions performed at the late stage revealed an inflammatory lesion (multifocal to coalescing suppurative sinusitis) with a very high density of intralesional fungal hyphae (Figure 9). No histological lesions were observed in the brain (not shown). Whether the disturbance in equilibrium may be caused by fungal infection of the inner ear cannot be excluded, but had not been investigated here. However, contrasting the decline in bioluminescence in infected lung tissues under cortisone acetate treatment, the steadily increasing bioluminescence from the sinus region might indeed resemble an increase of the fungal biomass.

Collectively, these results suggest that in the cortisone acetate condition, the early infiltration of neutrophils results in parenchymal destruction without stopping conidial germination. Three days post infection, neutrophils encircling *A. fumigatus *conidia and hyphae may limit fungal spread. However, despite the obvious killing of some fungal cells, these neutrophils are not able to completely prevent disease progression and mice suffer strongly from the severe inflammatory processes.

### RB6-8C5 treatment

To determine the effect of neutrophil depletion at specific time points in relation to infection, mice were treated with a single 0.1 mg intraperitoneal dose of monoclonal antibody RB6-8C5 (anti-Gr-1; anti-Ly6G/Ly6C). This method of transient neutrophil depletion was chosen because it is well characterized and specific compared with other methods (eg, administration of cyclophosphamide [17] or irradiation and results in more than 99% depletion in the circulation [22]. Treatment of mice with the anti-neutrophil antibody RB6-8C5 led to a high susceptibility of mice for IA (Figure 1B). However, the luminescence signal was significantly lower than that obtained for cortisone acetate treated mice and the highest values were obtained two days post infection, later than the day 1 peak observed in the cortisone acetate-treated group (Figure 1C).

#### Monocytes and macrophages are insufficient to prevent conidial germination and hyphal spread in the absence of neutrophils

**Figure 10:**
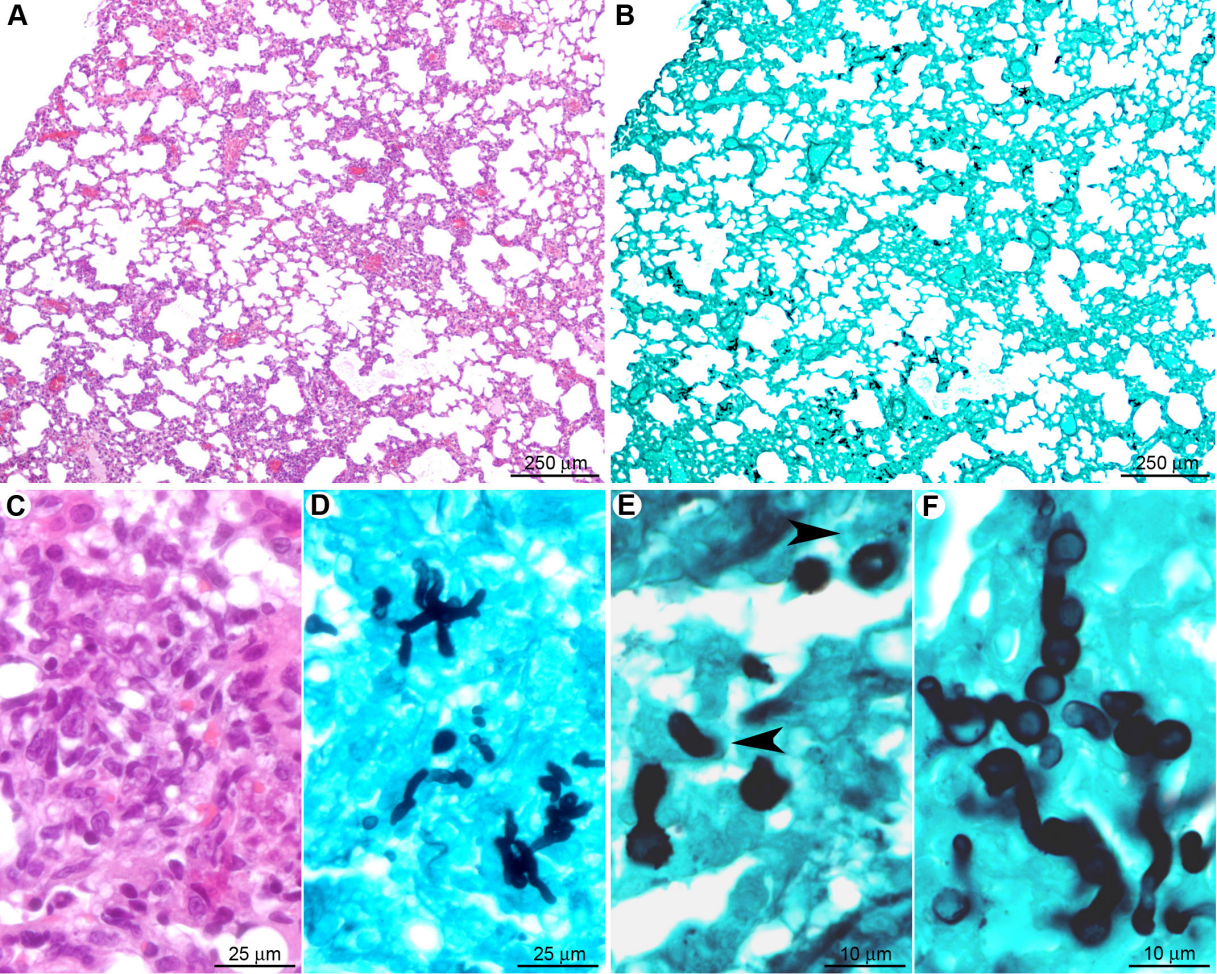
**In the early stage after RB6-8C5 treatment, although immunocompetent, macrophages were not sufficient to avoid conidial germination**. (A): Multifocal small inflammatory infiltrates randomly scattered in the pulmonary parenchyma. (B): Small clusters of fungi were observed in the inflammatory infiltrates. (C): Inflammatory infiltrates were located in alveolar spaces or interalveolar interstitial tissue. They contained mononucleated cells (mainly macrophages but also rare lymphocytes and plasma cells). (D, E, F): Early germinating conidia were observed in the inflammatory infiltrates either free or in the cytoplasm of alveolar macrophages (arrowheads). Note that the conidia and hyphae were less mature than under cortisone acetate treatment (Figure. 6). *A, C: HE staining; B, D, E, F: GMS staining*.

One day post infection in neutrophil-depleted mice (Figure 10), multifocal pulmonary lesions were observed, characterised by small infiltrates (surface less than 150 μm^2^) of mononucleated cells (mainly macrophages but also lymphocytes and rare plasma cells), located either in alveolar spaces or in interalveolar interstitial tissue (Figure 10A, C). Neutrophils were not observed within these lesions, indicating a successful depletion of this cell population by the RB6-8C5 treatment. Lesions represented 1.9 ± 0.5% of the parenchymal surface (Table 1). Germinating conidia and short hyphae were observed (Figure 10B, D-F) in extracellular spaces, typically surrounded by small clusters of inflammatory infiltrates (Figure 10D, F), or within the cytoplasm of AM (Figure 10E). In contrast to the cortisone acetate treated-mice, no difference in the fungal maturation stage was observed between intra-bronchiolar and intra-alveolar fungi, and fungi displayed less parenchyma infiltration potential.

**Figure 11:**
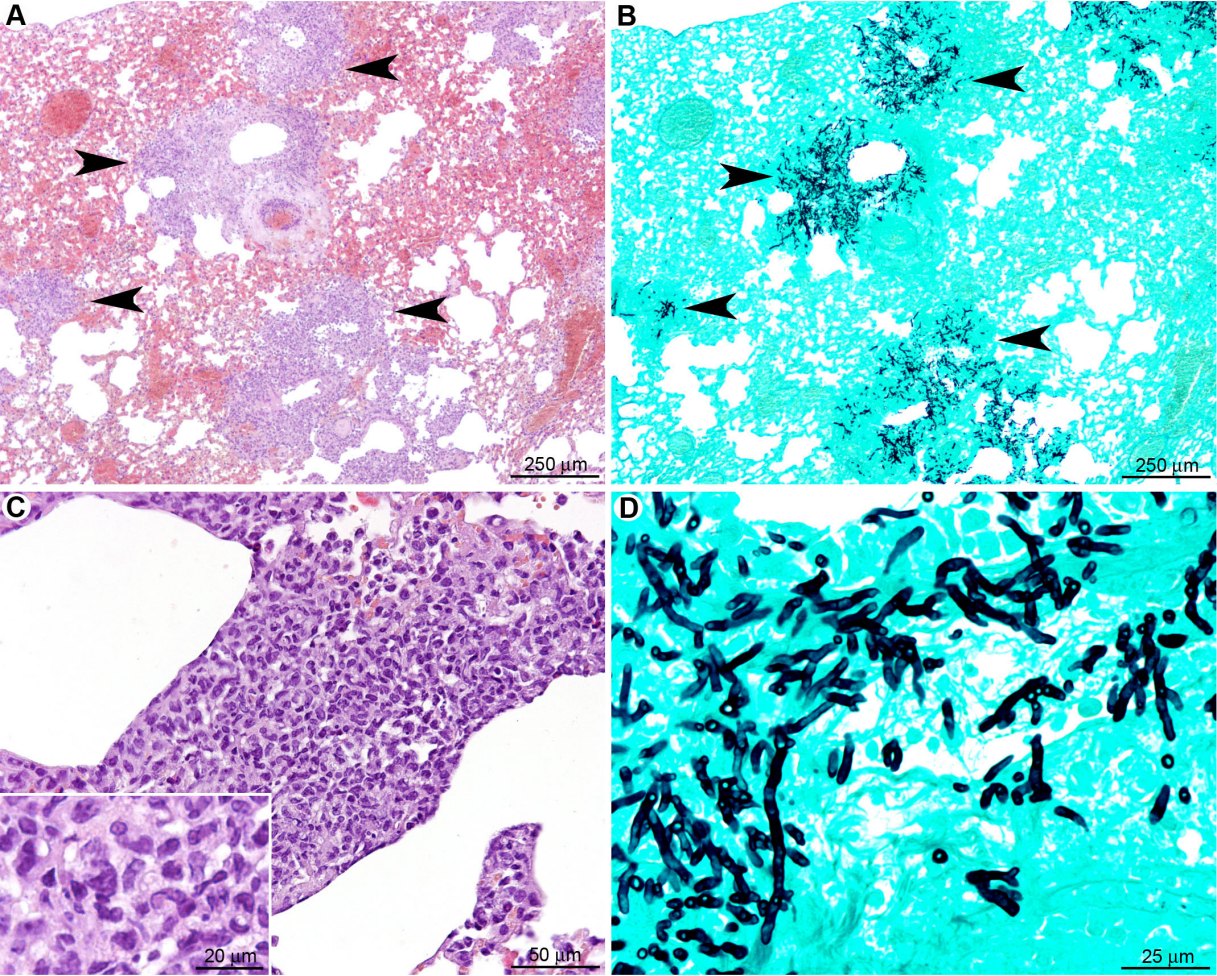
**In the late stage after RB6-8C5 treatment, macrophages and recruited monocytes were unable to prevent fungal lung colonisation**. (A): Multifocal large inflammatory infiltrates centred on bronchioles but extending to alveoli and blood vessels (arrowheads). (B): Fungi displayed a high infiltrative potential with a marked extension to alveoli (arrowheads). (C): Inflammatory infiltrates were composed of mononucleated cells; mainly macrophages (inlay). (D): Hyphae were mature and displayed a high invasive potential. *A, C: HE staining; B, D: GMS staining*.

The late stage (three days post infection) of IA induced by transient neutrophil depletion (Figure 11) was characterised by a multifocal inflammatory lesion, centered on bronchi and bronchioles but extending to alveoli and blood vessels as well (Figure 11A). The lesions were extensive, with large areas of necrosis and vascular involvement that was more pronounced than in cortisone acetate-treated mice (Table 1). Mature septated fungal hyphae were observed infiltrating bronchiolar and alveolar spaces as well as interstitial tissue (Figure 11B, D). Hyphae were more numerous than in cortisone acetate-treated mice and infiltrated the pulmonary parenchyma more readily (Figure 11A, B). The inflammatory infiltrate was predominately composed of mononuclear cells (monocytes/macrophages and lymphocytes and plasma cells) (Figure 10C). Individual lesions measured up to 500 μm^2 ^in area and accounted 18.9 ± 2.8% of the total lung section surface (Table 1), which is even higher than the area affected under cortisone acetate treatment.

Taken together, these data indicate that the recruitment of mononuclear cells, in the absence of neutrophils, is insufficient to prevent conidial germination, hyphal outgrowth and tissue infiltration. It is likely that the severe vascular and parenchymal lesions observed in RB6-8C5-treated mice prevented the development of high bioluminescent signals in vivo. This is most likely due to hypoxia resulting from the pulmonary parenchyma destruction, which was even more severe than under cortisone acetate treatment.

### Cyclophosphamide treatment

Treatment with cyclophosphamide was expected to cause severe neutropenia accompanied by a reduction of monocytes. However, resident alveolar macrophages were not expected to be affected by this treatment. Bioluminescence imaging revealed that cyclophosphamide treatment resulted in a delayed (apparent at day 2 to day 3 post-infection), but steadily increasing bioluminescence signal until mice succumbed to progressive disease (Figure 1C and Figure 2 inlet).

#### At the late stage of infection, in the absence of inflammatory cells, *A. fumigatus* disseminates rapidly in cyclophosphamide-treated mice

**Figure 12:**
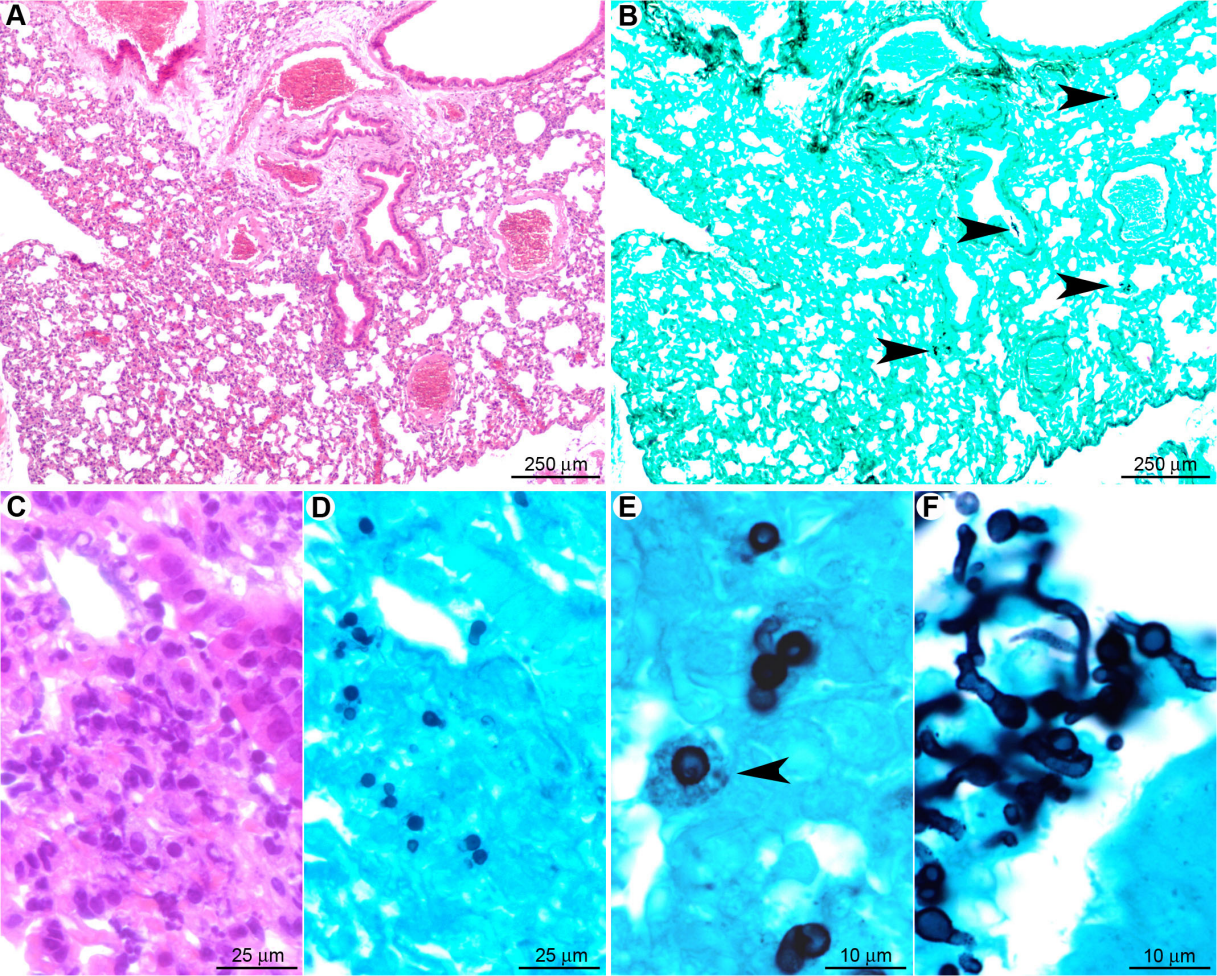
**In the early stage, *A. fumigatus *germination was delayed after cyclophosphamide treatment**. (A): At a low magnification, no significant histological lesion was observed. B: Only small clusters of conidia were multifocally detected (arrowheads). C. At a high magnification, only small infiltrates of neutrophils were noted in bronchiolar and alveolar spaces. (D): Non-germinated and early germinating conidia were observed in these inflammatory infiltrates. (E): Intra-alveolar conidia at a very early stage of germination (swollen conidia). Some conidia were observed in the cytoplasm of alveolar macrophages (arrowhead). (F): Intra-bronchiolar conidia were either swollen or started to form hyphae. Note that this stage of maturation is much less pronounced than observed in the early stage of cortisone acetate (Figure 6D) and RB6-8C5 treatment (Figure 9D). *A, C: HE staining; B, D, E, F: GMS staining*.

At day one post-infection (Figure 12), histopathology revealed no significant histological lesion but rare neutrophils could be observed in bronchiolar spaces (Figure 12A, C). Non-germinating and rare early-germinating conidia were detected throughout bronchiolar and alveolar spaces (Figure 12B, D). As in the cortisone acetate-treated mice, intrabronchiolar fungi (Figure 12F) were seen at a more advanced stage of maturation than intra-alveolar fungal cells (Figure 12E). However, hyphal branching was rarely observed at the early stage, even in intrabronchiolar regions (Table 1), confirming the data from the quantitative analysis of the fungal DNA from infected lungs, which implied, despite the small animal group studied, that conidia germination is delayed under cyclophosphamide compared to the cortisone acetate treatment (Figure 2).

**Figure 13:**
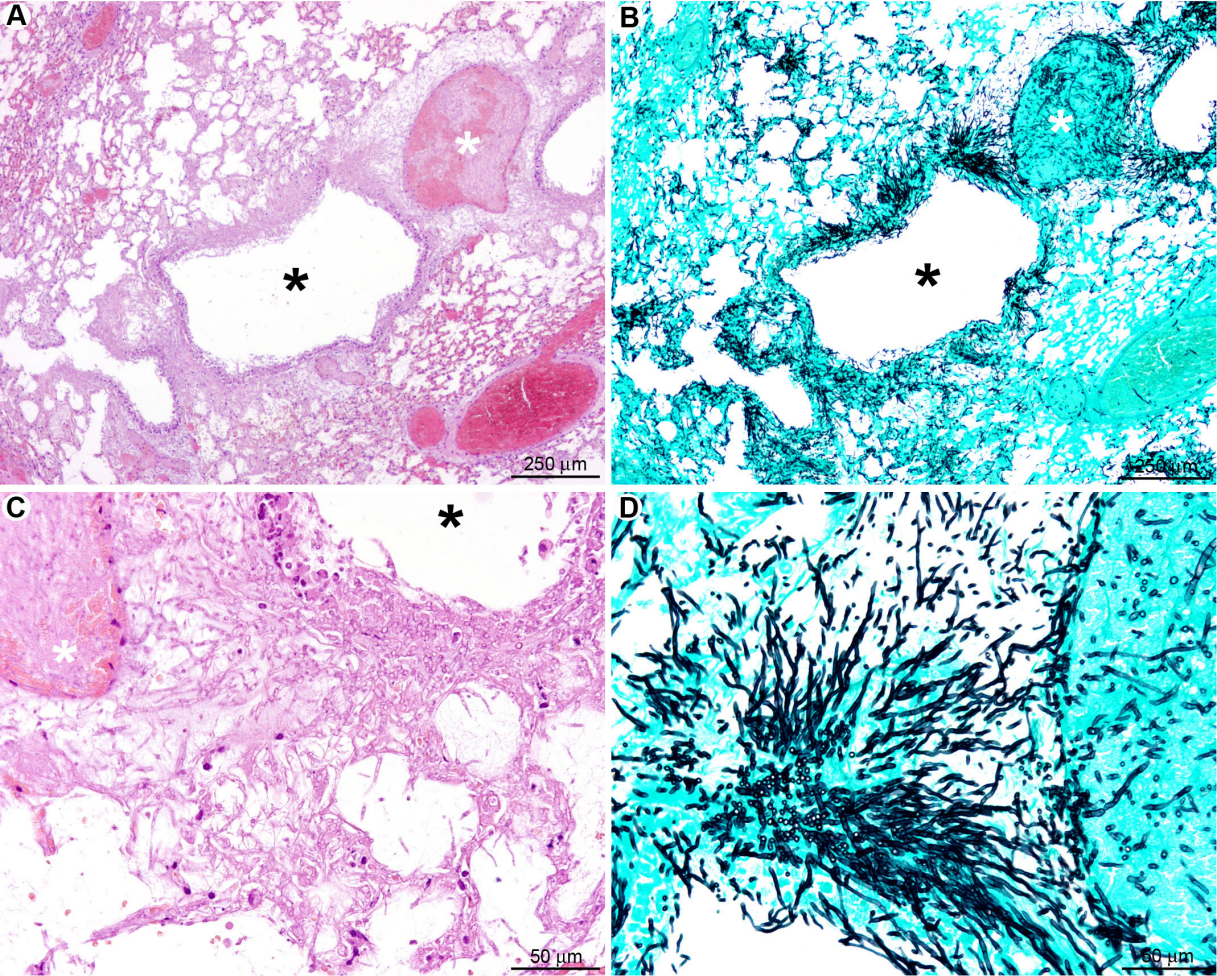
**In the late stage after cyclophosphamide treatment no inflammatory response was observed and *A. fumigatus *rapidly colonised the pulmonary parenchyma**. (A): Diffuse lesion characterised by a total absence of inflammatory response with a severe destruction of the bronchoalveolar structures (black star: bronchiole; white star: pulmonary artery). (B): Severe parenchyma colonisation by the fungus with infiltration of bronchioles (black star) as well as pulmonary arteries (white star). (C): Destruction of the bronchiolar (black star), alveolar, and vascular (white star) walls by hyphae. (D): Branched mature hyphae were observed, displaying a high infiltrative potential. *A, C: HE staining; B, D: GMS staining*.

In contrast, the late stage of pulmonary infection (Figure 13) was characterised by a severe and diffuse destruction of bronchoalveolar structures (Figure 13A), without any inflammatory cell infiltrate (Table 1). The parenchyma destruction was due to severe fungal parenchymal and vascular wall infiltration, leading to thrombosis and infarcts (Figure 13B). Bronchial, bronchiolar, and alveolar epithelial cells were necrotic (Figure 13C). Grocott methenamine silver staining showed a high number of mature septated fungal hyphae, spreading diffusely from bronchiolar spaces to alveoli and infiltrating blood vessels (Figure 13D), as already assumed from the increasing bioluminescent signal and the high amount of fungal DNA obtained from these tissues (Figure 2). Collectively these results demonstrate that immune effector cells recruitment is vital to limit hyphal growth and dissemination.

## Discussion

In this study we successfully imaged murine invasive pulmonary aspergillosis using bioluminescence recordings in a serial manner. We applied different immunosuppression regimens to elucidate their impact on the susceptibility of mice to invasive aspergillosis (IA). By combining bioluminescence imaging and histopathology we gained new insights on the impact of different immune effector cells (mainly macrophages and neutrophils) in host defense against conidial germination and tissue invasion.

Interestingly, under conditions of high inflammation, such as the cortisone acetate or RB6-8C5 treatment, bioluminescence signal intensities nicely reflected the early germination of conidia, but only showed limited correlation with the amount of alive fungal cells at later time points of infection. Quantification of the fungal DNA from late time points of cortisone acetate treated animal implied that the number of living cells stayed constant over time. This result confirmed that neutrophils, although affected in their killing capacity by the corticosteroid, limited the uncontrolled spreading of fungal mycelium through the lung tissues. However, one would have expected that the bioluminescence signal stays at a high level rather than declining. Due to the large necrotic areas (covering approximately 11% of the whole lung parenchyma), we attribute the decline of the bioluminescence signal to the development of hypoxia, as observed in tissues after stroke or myocardial infarction and for growing tumors, which become hypoxic when they outgrow the vascular supply [23]. The limitation of bioluminescence imaging in hypoxic tissues has already been described by investigating the decrease in bioluminescence of luciferase-transfected gliosarcoma tumor cells under defined hypoxic in vitro conditions [24]. Additionally, bioluminescent implanted tumor cells can become necrotic at a certain age with subsequent decline of bioluminescence although the tumor itself does not reduce its size [25]. This latter scenario is likely to be comparable to our results obtained during bioluminescence imaging of invasive aspergillosis under cortisone acetate and RB6-8C5 antibody treatment. In addition, the occurrence of hypoxia has been assumed from the attenuated virulence of *A. fumigatus *mutants with a defective adaptation to hypoxic conditions [26] and seems confirmed independently by our bioluminescence measurements. Although the correlation of the bioluminescent signal at later time points with the fungal burden is limited the decline in bioluminescence under in vivo conditions in late stages of infection acts as an indicator of tissue destruction and hypoxia and indicates the imminent death of the animals.

Despite the low correlation of fungal biomass and bioluminescence at late time points after infection in the cortisone acetate and RB6-8C5 treatments, a good correlation between the increase in the fungal biomass and the bioluminescence was observed under the cyclophosphamide regimen. Under this treatment, although the growing hyphae were responsible for diffuse parenchyma lesions, the accessibility of oxygen remains possible in the absence of inflammation. At late time points, an ongoing increase of the luminescence signal reflects the increase of biomass. Therefore, cyclophosphamide immunosuppression seems best suited to follow the effect of antifungal drug treatment on clearance of fungal infections.

This study additionally allowed gaining new insight concerning the impact of different immune effector cells in the defense against invasive aspergillosis. Alveolar macrophages (AM) were assumed to play an important role in clearance of conidia from tissues and provide a "first-line of defense" against *A. fumigatus *infections [3]. AM are thought to trigger the recruitment of immune effector cells to the site of infection after recognition and phagocytosis of conidia [27] through the release of inflammatory and chemotactic mediators.

Due to the importance of AM in conidial host defense, we expected that their reduction by the clodrolip treatment would increase the susceptibility of mice to IA. This assumption was not confirmed experimentally. Intranasally clodrolip-treated mice showed a 80% reduction in the number and viability of AM [28,29], but a 2.6 fold increase in the number of BAL fluid neutrophils, one day post-infection. A significant increase in the neutrophil number in BAL fluid of macrophage-depleted (clodrolip-treated) mice 24 hours after instillation of *Pseudomonas aeruginosa *has already been reported. However, in this work macrophage-deficient mice showed impaired bacterial clearance [30]. In contrast, in our model, neutrophil migration into the airways of macrophage-depleted infected mice is likely to have prevented conidial germination per se.

Supporting this idea, we found that the thoracic region or BAL fluid of AM-depleted animals only showed a slight increase in bioluminescence above control levels (Figure 3). This finding correlated with survival data and histopathological findings, demonstrating an absence of conidial germination in AM-depleted mice. These results do not fully exclude the possibility that (i) there were still sufficient residual viable alveolar macrophages to trigger an immune response due to only 80% of depletion and (ii) the possibility of recruitment of peripheral monocytes or (iii) that the increase in recruited neutrophils was able to control the infection in the absence of normal alveolar macrophage counts. The first and second scenarios, however, appear rather unlikely, because hardly any macrophages or monocytes were observed in histopathologic analyses at day one after infection. The third scenario appears quite likely, because histopathological analysis revealed a strong infiltration of neutrophils encasing ungerminated conidia. In contrast, functionally attenuated neutrophils and macrophages in corticosteroid-treated mice allowed development of invasive disease despite robust cellular recruitment in the lung parenchyma.

The treatment of mice with cortisone acetate or the combination of clodrolip and cortisone acetate led to 100% mortality and invasive fungal growth within the lung tissue. Although systemic administration of corticosteroids increases the number of circulating neutrophils by three- to fivefold [31], their ability to damage *A. fumigatus *hyphae is strongly reduced [32]. One day post-infection, the lung tissue showed an extensive neutrophilic infiltration that surrounded germinating conidia. These neutrophils were able to delay uncontrolled tissue invasion by killing some proportion of fungal hyphae. As a consequence of the neutrophil infiltration severe tissue damage accompanied by parenchymal destruction (necrosis) was observed, leading to a decreased bioluminescence as described above. It is also noteworthy that under cortisone acetate treatment the efficiency of alveolar macrophages in inhibiting conidial germination after phagocytosis was strongly defective. None of the other treatment groups yielded hyphal germlings as early as one day post-infection. It could be assumed that this rapid germination is due to growth stimulation via *A. fumigatus *corticosteroid receptors [33]. However, experiments, in which different concentrations of cortisone acetate were added to *A. fumigatus *cultures, neither stimulated conidia germination, nor increased the light emission (data not shown). Since cortisone acetate itself constitutes an "inactive" corticosteroid derivative, which is converted into "active" cortisol during metabolism in the liver [34], it might be possible that a stimulation of germination is only mediated by this metabolite rather than by cortisone acetate.

Another possibility for the rapid germination of conidia is given by a neutrophil mediated tissue destruction releasing large amounts of nutrients from tissue cells, which enhanced the germination speed under this immunosuppresive regimen. The mild inflammation under RB6-8C5 treatment one day post infection and the absence of inflammation under cyclophosphamide treatment may not provide the same nutritional conditions leading to a delayed germination when compared to the cortisone acetate treatment.

Another piece of evidence that supports the dependence on the number and functional integrity of neutrophils in the clearance of *A. fumigatus *is the observation that RB6-8C5 treatment renders mice highly susceptible to IA. Our results confirm earlier studies [17,35,36,22] and clearly demonstrate that AM and monocytes cannot functionally compensate for loss of neutrophils. RB6-8C5 treated mice succumbed to IA with a similar time course as cortisone acetate-treated mice. However, a notable difference between both models was the absence of neutrophils and the severe tissue infiltration by mononuclear cells (mainly macrophages) seen in RB6-8C5-treated mice at days three to four after infection. This tissue infiltration covered approximately 19% of the total lung surface and was more severe than observed in the cortisone acetate treatment group (approximately 11%).

Treatment with cyclophosphamide was assumed to have the strongest impact on the development of IA. It results in: (i) a reduction in the number of monocytes and neutrophils in the peripheral blood by 64 and 88%, respectively [37-39], (ii) a reduction in the number of AM and neutrophils in an experimental lung infection with *Streptococcus pneumoniae *[40], (iii) an impairment of phagocytosis [41], (iv) an immune dysfunction through reactive oxygen intermediate-induced damage to the immune system cells [42-44] without alteration of the degranulation process [38] and finally (v) a failure in neutrophil chemotactic function [45]. As expected, under this treatment, we did not observe inflammation within the infected tissues. Therefore, mice treated with cyclophosphamide succumb to uncontrolled infection resulting in tissue destruction and blood vessel infiltration by the fungal mycelium and the fungal biomass produced under this regimen was by far most pronounced at late time points (Figure 2 and 13). In contrast, cortisone acetate and RB6-8C5 treatment likely results in additional tissue injury due to the strong, but ineffective host inflammatory response.

Interestingly, the luminescence additionally enabled us to detect and monitor extrathoracic growth of *A. fumigatus *in particular in the sinus area even in cortisone acetate treated mice. The resulting suppurative sinusitis may indicate a defect in the innate immune response in the upper respiratory airway rather than dissemination.

Reflecting on the outcome of aspergillosis from the different infection models, we conclude that AM are likely to be important in orchestrating the early immune response to recruit other immune effector cells. However, although able to slow fungal outgrowth, AM are insufficient to clear the infection in the absence of neutrophils. Neutrophil depletion by the RB6-8C5 antibody leads to a predominately monocyte infiltration to the site of infection. Influx of mononuclear cells is insufficient to replace neutrophil function. Corticosteroid treatment leads to the most rapid germination of conidia, which may reflect functional inactivation of alveolar macrophages followed by the ongoing influx of neutrophils, which are attenuated in their conidial and hyphal killing mechanisms. Although after corticosteroid-treatment, neutrophils were not able to inactivate growing hyphae completely, they retarded hyphal tissue invasion at the great cost of massive tissue inflammation and necrosis. Therefore, preservation of neutrophil number and function is indispensable for the control and clearance of *A. fumigatus *infections. Macrophages may play an important role in orchestrating the immune response, but their action alone is not sufficient to combat *A. fumigatus*.

Our data suggest that the early neutrophil recruitment is crucial to form an efficient immune response against *A. fumigatus*. This assumption is supported by two previous studies, which have reported that mice deficient in the chemokine receptor CXCR2 (CXCR2-/- mice) display a defect in neutrophil recruitment and were more susceptible to IA [36,35]. Therefore, we conducted a preliminary investigation, in which we used a bioluminescent *A. fumigatus *strain to monitor the pathogenesis of CXCR2-/- mice. This experiment revealed an overall average of 3-fold increase of bioluminescence signal within the thoracic region of knockout compared to wild type mice. At day 6 post infection, a 12 fold-increase in luminescence was observed in knockout animals with a mortality rate of more than 60%, whereas all immune competent wild-type mice survived (data not shown). Although this experiment has to be confirmed by characterizing the histological lesions, it fits well with the assumption that the early recruitment of immunocompetent neutrophils is one of the most important factors to combat the initial onset of invasive aspergillosis.

## Conclusions

Taken together, the bioluminescent *A. fumigatus *strain provides a valuable tool to define the progressive nature of IA under different immunosuppressive regimens, although the quantification of fungal biomass by bioluminescent imaging was difficult to assess especially under inflammatory conditions. However, in order to confirm that the tendency of the progression of infection is correctly assigned by bioluminescence imaging, we confirmed our results by histopathologic analysis and quantification of the fungal DNA by qRT-PCR. The latter method is the most reliable measure for quantification of living fungal cells, but cannot be used in time response analyses since the animals need to be sacrificed to gain the infected organs. Although larger animal groups and all immunosuppression regimens need to be investigated by quantitative real-time PCR, it appears that bioluminescence imaging cannot be used for replacing alternative methods for quantification if an exact value for fungal biomass in a certain animal and time point needs to be determined. This is mainly due to the fact that bioluminescence does not increase or decrease linearly with the burden as determined by quantitative real-time PCR since determination of light emission from living animals is strongly dependent on availability of oxygen. However, and most importantly, bioluminescence imaging was indeed able reflecting the general tendency of the speed of fungal germination, tissue invasion and inflammatory response. Histopathology revealed a rapid germination of conidia under cortisone acetate treatment and, coinciding, a high bioluminescent signal was obtained. At later stages, neutrophils partially inactivated fungal mycelium and caused tissue necrosis under corticosteroid treatment. In agreement, the bioluminescent signal strongly declined. Contrarily, under cyclophosphamide treatment conidia germination is delayed. Therefore, one day after infection only a weak bioluminescence signal was detected. However, at later time points under this regimen, a strong fungal invasion of the lung parenchyma was observed in histopathology and confirmed by quantification of fungal DNA. Coinciding, the bioluminescence strongly increased. Therefore, bioluminescence signals cannot be used for comparison of the fungal burden among different immunosuppression regimens but within one well-defined regimen, the bioluminescence correlates well with the independently determined fungal germination speed, immune response and the fate of fungal cells within the infected tissue.

By using the bioluminsescence imaging system, we found that experiments that perturb the number, recruitment, and function of neutrophils result in predictable patterns of invasive aspergillosis that can be imaged serially in real time with bioluminescence imaging. In vivo monitoring shows light emission from lungs as soon as 24 hours post infection, indicating rapid outgrowth of the fungus. Therefore, early diagnosis of fungal infections is of tremendous importance. In addition, our study provides new insights into the innate immune response emphasizing an essential role for neutrophils as recruited phagocytes in the early innate response to *A. fumigatus*.

The currently constructed strain seems most suitable for disease monitoring in host system that have undergone myeloablation (e.g. cyclophosphamide treatment). The reproducible imaging results from small groups of animals and is likely to help in substantial cost savings in trials that examine the effects of pharmaceutical compounds, antibodies, and genetic or cellular lesions in small animal models of IA. In further studies, bioluminescence imaging will be used to assess the efficacy of antifungal drugs under in vivo conditions. A successful monitoring of clearance of fungal infections might help improving future treatment strategies for combating invasive fungal infections.

## Methods

### Strain culturing and mouse infection

#### *A. fumigatus *strain C3

The bioluminescent *A. fumigatus *strain C3 [16] was used in all experiments and was subcultured on 2% malt extract agar slants for 8 days at room temperature. Conidia were harvested by scrapping them from the slant culture with 2 ml of phosphate buffered saline supplemented with 0.1% Tween 20 (PBST). The suspension was filtered through a 40 μm cell strainer (BD Falcon, Bedford MA, USA) to separate conidia from contaminating mycelium.

#### Mice

Male BALB/cJ mice (25 g, 8 week-old) were supplied by the Centre d' Elevage R. Janvier (Le Genest Saint-Isle, France). Mice were fed with normal mouse chow and water *ad libitum *and were reared and housed under standard conditions with air filtration. Mice were cared for in accordance with Institut Pasteur guidelines in compliance with the European animal welfare regulation. Prior to intranasal infection one of the following immunosuppression regimens was applied:

#### (i) Cortisone acetate treatment

Cortisone acetate was suspended in sterile phosphate buffered saline (PBS) to give a final concentration of 125 mg/ml. The suspension was sonicated at 37°C for at least 30 min to prepare a homogenous suspension. Immunosuppression was performed as described previously [46], whereby mice were immunosuppressed with two single doses of 25 mg cortisone acetate (Sigma Aldrich, St Louis, MO), which were injected intraperitoneally three days before and immediately prior to infection with conidia (day 0).

#### (ii) RB6 purification and treatment

The RB6-8C5 anti-neutrophil antibody was purified from ascites (gift from Robert Coffman, DNAX Corp.) by chromatography over a HiTrap protein G column (1 ml bed volume, GE Healthcare, Freiburg, Germany). Aliquots containing 500 μg of purified antibody in PBS were shock-frozen in liquid nitrogen and stored at -80°C until use. For depletion of neutrophils, each mouse received 100 μg of RB6-8C5 antibody (150 μl) injected intraperitoneally one day prior to infection.

#### (iii) Cyclophosphamide treatment

For bone marrow stem cell depletion, cyclophosphamide was injected intraperitoneally (200 mg/kg) four and one day prior to infection. The cyclophosphamide injection was repeated every other day post-infection.

#### (iv) Clodrolip treatment

Clodronate liposomes (Clodrolip) were prepared as described previously [47,48]. Clodronate was a gift of Farchemia, Treviglio, Italy. The liposomes act as carriers for clodronate, which is toxic for phagocytic cells. Two days prior infection, a volume of 83 μl containing 1.5 mg of Clodrolip was directly instilled into the nares of anesthetized mice to deplete alveolar macrophages. Mice instilled with empty liposomes were used as controls.

Additionally, certain mice received both clodrolip and cortisone acetate. This regimen included one dose of cortisone acetate and clodrolip at day -3, clodrolip alone at day -2 and cortisone acetate alone at the day of infection.

### Mouse infection

Mice were anesthetised by an intramuscular injection of 0.1 ml of a solution containing 10 mg ketamine (Imalgène 1000, Merial, Lyon, France) and 0.8 mg xylazine (Bayer, Leverkusen, Germany) per mouse. 2 × 10^6 ^conidia in 25 μl of PBS 0.1% Tween 20 were applied to the nares of the mice. Deep anaesthesia ensured inhalation of the conidial inoculum. Infected mice were daily monitored by bioluminescence imaging using an IVIS 100 system (Xenogen Corporation, Alameda, CA, USA). Weight loss was monitored at 24 h intervals starting from day -4. In all experiments, mice were kept for a maximum of 15 days post infection.

### Bronchoalveolar lavages

Bronchoalveolar lavage (BAL) fluid was harvested as previously described [20]. Mice were euthanized by injection of Pentobarbital (Sanofi Santé Animale, Libourne, France) and the respiratory tract was exposed by dissection. A small incision was made near the top of the trachea, and a blunt-end 20-gauge needle was inserted and tied in place with surgical thread around the trachea. BAL fluid was obtained by 10 rounds of filling the lungs with 0.7 ml PBS and withdrawing as much of the liquid as possible. The samples were centrifuged to collect BAL fluid cells. BAL fluid cells were washed and resuspended in 1 ml PBS and aliquots were removed for counting with a hemocytometer and for cytospin centrifugation on a microscope slide, followed by DNA staining with Hoechst 33342 for identification of cell types. To determine the numbers of macrophages and neutrophils in the samples, 100 cells from several microscopy fields were identified.

Flow cytometry using macrophage marker antibodies F4/80 (Miltenyi-Biotec, Bergisch Gladbach, Germany) and Gr-1 (Biolegend, San diego CA USA) was used to verify the extent of macrophage depletion within the BAL of clodrolip treated animals. Cell viability was evaluated using the trypan dye exclusion (Sigma-Aldrich).

### In vivo and in vitro imaging of bioluminescence

Images were acquired using an IVIS 100 system according to the manufacturer's instructions and as previously described [16]. In brief, 100 μl of PBS containing 3.33 mg D-luciferin was intraperitoneally injected in mice before each measurement. Mice were anesthetized using a constant flow of 2.5% isofluorane mixed with oxygen using an XGI-8 gas anesthesia system (Xenogen Corporation). Images from mice were acquired 10 min after luciferin injection. Acquisition and quantification were performed using Living Image software version 3.1 (Xenogen Corporation). Quantification of photons per second emitted by each organ was performed by defining regions of interest corresponding to the respective organ of interest. The presence of *A. fumigatus *within the different organs was confirmed by histopathological analysis.

For *in vitro *measurement of fungal germination within the BAL, D-luciferin in a final concentration of 10 mM was added directly to cells pelleted at the surface of chamber slides. The reaction was pre-incubated for 10 min at room temperature and measurement was performed with the IVIS 100 system.

### Determination of fungal DNA from infected lungs by quantitative real-time PCR

A quantitative real-time PCR approach was selected to determine the fungal burden by quantification of the amount of fungal DNA among the total DNA isolated from lung tissues. The lung of a mouse not infected with *A. fumigatus *served as negative control. Four mice either immunosuppressed with cortisone acetate or with cyclophosphamide were infected intranasally with 2 × 10^6 ^conidia of the bioluminescent strain C3. From each group two were sacrificed on day 1 after infection (early time point) and two mice at day 3 (late time point). The control mouse was sacrificed on day three. Bioluminescence at the early time point was measured from alive animals, whereas at the late time point bioluminescence was additionally recorded from explanted lungs by direct injection of D-luciferin. Lungs were cut into small pieces and briefly washed in phosphate buffered saline. Excess liquid was removed on paper tissues and the weight of lungs was determined. The complete lung from each animal was frozen in liquid nitrogen and ground to a fine powder. Approximately 100 mg of each powdered lung was used for DNA extraction via the MasterPure yeast DNA extraction kit (Epicentre Biotechnologies, Biozym Scientific GmbH, Hessisch Oldendorf, Germany) as described in the manufacturer's protocol. As a slight modification and for obtaining DNA of higher purity grade, an ethanol precipitation step of the DNA was included. The amount of DNA extracted from the lung tissues was quantified by a NanoDrop spectrophotometer. All samples were diluted to 100 ng/μl and quantified again to confirm the DNA concentration of each sample. As a standard for quantification of the amount of fungal DNA among the total DNA extracted from lung tissues, *A. fumigatus *genomic DNA was isolated by the same procedure from a culture grown for 20 h on minimal medium containing glucose (50 mM) and peptone (0.5% w/v) as nutrient sources. The TaqMan quantitative real-time PCR approach used based on the standard operation procedure (SOP) described elsewhere http://www.sacmm.org/pdf/Determination%20of%20Tissue%20Fungal%20Burden%20utilizing%20Quantitative%20Real%20Time%20PCR.pdf. The TaqMan^® ^Universal PCR Master Mix (Applied Biosystems, Darmstadt, Germany) was used in all approaches. In brief, the genomic DNA region coding for the 18S rRNA from *A. fumigatus *was used as the target for amplification and quantification of fungal DNA. A specific probe containing a 6-FAM-phosphoramidit labeling at the 5'-end and a TAMRA labeling at the 3'-end was used for detection of the amplification products. Amplification was performed on a StepOnePlus Real-Time PCR system (Applied Biosystems) and data were evaluated by using the StepOne software version 2.0 (Applied Biosystems). The standard curve on genomic DNA from *A. fumigatus *was generated from three technical replicates, whereby each replicate contained 6 dilutions in the range between 100 and 3.125 ng per reaction (stability index of standard curve = 0.99). The amplification program consisted of an initial denaturation at 95°C for 10 min followed by 40 cycles with denaturation for 15 s at 95°C, annealing for 30 s at 54°C, and amplification for 30 s at 72°C. All DNA samples from lung tissues were measured from 3 dilutions (from 500 to 125 ng total DNA per reaction) in two technical replicates. All six data points from each sample were used for calculation of the amount of fungal DNA per μg of total DNA.

### Histopathology

For the histopathological analysis, a group of five mice was studied at each time point (early and late time point), for each immunosuppressive condition. After necropsy, organs of interest (lung, nasal sinus, and brain) were immediately fixed in 4% neutral-buffered formalin and embedded in paraffin. Mouse skull and sinus histological analyses required decalcification in a solution of 4% buffered formalin and 10% trichloroacetic acid for approximately 2 months. Five μm sections were cut and stained with hematoxylin and eosin (HE) and Grocott's methenamine silver (GMS, for detection of fungi) [49]. The lesion profiles were very similar between mice of the same group.

The presence of conidia and hyphae were quantified as evaluated in general in histology within tissue thin sections. This semiquantitative fungal burden is presented as follow: - none, +/- minimal, + mild, ++ moderate, +++ marked, ++++ severe.

The total surface of inflammatory cell infiltrates in tissue sections was measured by morphometric analysis in 22 to 40 microscopic fields, covering an entire lung section for each animal, at 4× magnification. Three mice were analyzed for each immunosuppressive condition. ImageJ 1.38× software (National Institute of Health, USA) was used for this analysis. Reliability was assessed by 20 repeated measurements over several days (coefficient of variation: 1.6%).

### Statistical analysis

All experiments were performed at least in triplicate with groups of 5 mice for each treatment. Comparisons between multiple groups were performed using one-way ANOVA. Significance between groups was determined with the Fisher's Least Significant Difference post hoc test. A p value of < 0.05 was considered statistically significant. Data are reported in the figures as means ± standard deviation.

## Authors' contributions

OI-G conceived and designed the experiments, carried out the fungal strain cultures, the animal and bioluminescence experiments, analysed the data and drafted the manuscript. GJ carried out the histopathology analysis and has been involved in the drafting and revising the manuscript. TMH has been involved in the conception and design and drafting and revising the manuscript. SD-B participated to the histopathology analysis, FP carried out the animal experiments, OYK analysed the data, MA-C carried out the cell data analysis, RS provided reagents, J-MC substantially contributed to the design and in the revision of the manuscript and MB conceived and designed the experiments, engineered the fungal strain, assisted in animal experiments, quantified the fungal burden by qRT-PCR, and drafted the manuscript. All authors read and approved the final manuscript.
